# Differential expression and regulation of *FASLG* by miR-5195/miR-3941 in age-related hearing loss

**DOI:** 10.1371/journal.pone.0331661

**Published:** 2025-09-19

**Authors:** Jeongmin Lee, Junseo Jung, Hyunsook Kang, Kyeongjin Park, Jong Bin Lee, Seongjun Choi

**Affiliations:** 1 Department of Otolaryngology-Head and Neck Surgery, Cheonan Hospital, College of Medicine, Soonchunhyang University, Cheonan, Republic of Korea; 2 Department of Biomedical Science, Soonchunhyang University, Asan, Republic of Korea; 3 Department of Otorhinolaryngology-Head and Neck Surgery, College of Medicine, Konyang University, Daejeon, Republic of Korea; University of Vermont College of Medicine, UNITED STATES OF AMERICA

## Abstract

Presbycusis, or age-related hearing loss (ARHL), is a progressive condition that involves a steady decline in auditory function, primarily caused by the physiological alterations that occur with aging. This disorder arises from the combined effect of multiple interconnected factors that progressively affect the auditory system over time. Genome-wide association studies (GWAS) and transcriptomic analyses in human populations are valuable approaches for identifying potential genes associated with ARHL. This research seeks to assess the potential of protective drugs or strategies for treating ARHL by analyzing target gene-miRNA interactions identified in human blood and associated with presbycusis. We performed RNA sequencing to analyze the transcriptomes of peripheral blood leukocytes from ARHL patients. To identify genes associated with ARHL, the RNA-sequencing data from the peripheral blood leukocytes were compared and further validated by real-time polymerase chain reaction (RT-qPCR) using whole blood samples from the same ARHL patients. To explore the involvement of target genes and microRNAs (miRNAs) in ARHL, we examined miRNA expression patterns using RT-qPCR and reporter gene assays. We found that four genes were up-expressed in ARHL serum: Fas Ligand (*FASLG*), Neural Cell Adhesion Molecule 1 (*NCAM1*), Nectin Cell Adhesion Molecule 1 (*NECTIN1*), Macrophage Receptor with Collagenous Structure (*MARCO*). The up-expressed *FASLG* is associated with cell apoptosis and aging. Additionally, we showed that miR‑5195 and miR-3941 regulated *FASLG* expression in House Ear Institute-Organ of Corti 1 (HEI-OC-1) and HeLa cells via targeting of *FASLG* using luciferase reporter assays. Finally, the over-expression of the *FASLG* gene may be associated with the development of ARHL, and the inhibitory role of miR-5195 and miR-3941 could be a key factor in the prevention or protection against ARHL.

## 1. Introduction

Age-related hearing loss (ARHL) is a multifactorial condition caused by the cumulative impact of aging on the auditory system. ARHL is the most prevalent chronic sensory impairment among older adults, with nearly half of individuals in their seventies experiencing hearing loss significant enough to interfere with communication [1]. ARHL is a multifactorial condition arising from the interplay of intrinsic factors, such as genetic predisposition, and extrinsic influences, including noise exposure and medication, which cumulatively impact the inner ear over a lifetime [2]. Animal models of ARHL have extensively documented the irreversible loss of hair cells in the organ of Corti (oC), which is widely recognized as a key factor contributing to ARHL [3]. Recently, there is a need to better understand the role of systemic immune aging in the mechanisms of ARHL and to develop preventive strategies against ARHL. ARHL appears to reflect the impact of various inflammation pathways associated with aging, both in the inner ear and systemically [4]. Macrophages, key elements of the innate immune system, are vital in the cochlear tissue damage associated with ARHL [5,6]. Therefore, we focused on the change of systemic immune response and peripheral blood leukocytes in ARHL patients.

Genome-wide association studies (GWAS) are actively advancing the understanding of genetic mechanisms underlying ARHL, leading to deeper insights into its pathophysiology. According to research by Wells et al., GWAS of adult hearing was conducted using data from over 250,000 samples in the UK Biobank [7]. The study identified 44 distinct genomic loci associated with hearing, including 34 loci that represent novel associations not previously documented. Additionally, a study utilizing data from the UK Biobank and several large-scale cohorts analyzed 125,749 cases of hearing loss and 469,497 controls, identifying 53 significant genetic loci. This research proposed new genes associated with hearing loss, such as COL9A3 and TMPRSS3, and highlighted the impact of both common and rare variants on the risk of hearing loss [8]. These studies leverage large-scale genomic data to uncover numerous genetic variants and loci associated with ARHL, exploring how genetic susceptibility influences ARHL onset. RNA sequencing analyses in ARHL have also identified 11 relevant genes, highlighting critical targets for developing future preventive and therapeutic strategies.

Fas cell surface death receptor (Fas) showed a significant difference and is a gene encoding the Fas ligand (*FASLG*). *FASLG* belongs to the tumor necrosis factor receptor superfamily and is primarily investigated as a ligand for Fas [9,10]. Binding of *FASLG* to the Fas receptor triggers apoptosis in cells [11,12]. When the Fas receptor is activated by *FASLG*, the receptor's death domain (DD) associates with Fas-Associated protein with Death Domain (FADD), facilitating their binding. This binding triggers the creation of the Death-Inducing Signaling Complex (DISC) within the cell. Caspase 8 activation is triggered via FADD. The formation of the DISC was initiated by the interaction between FADD and caspase 8. As the level of active caspase-8 rises, it triggers the activation of caspase 3. In another subtype, apoptosis is initiated by proteins of the B-cell lymphoma 2 (BCL-2) family, such as BH3 Interacting Domain Death Agonist (Bid), which promote the release of cytochrome C from mitochondria. This process is driven by the depolarization of the mitochondrial membrane [9,13–15].

MicroRNAs (miRNAs) are small non-coding RNAs, typically 20–24 nucleotides long, that regulate gene expression [16,17]. MiRNAs regulate gene expression through their interaction with the complementary 3’ untranslated region (3’UTR) of target mRNAs [18]. MiRNAs are essential in regulating numerous biological processes, and it has been suggested that miRNAs are present in the cochlea, where they are linked to ARHL [19–21]. MiRNAs, which were discovered in the past decade, have been shown to play significant roles in various disease mechanisms, including those related to the inner ear. These small RNA molecules influence physiological, developmental, and pathological processes in the cochlea by regulating the translation or expression of specific target mRNAs [22]. Additionally, miRNAs are present and remain stable in the bloodstream, where they can be encapsulated within membrane-bound vesicles [23,24]. Circulating miRNAs may play a role in cell-to-cell communication, secretion, and various other cellular processes, including cell death [25].

The exact role of miRNAs in the pathogenesis of ARHL is not yet fully clarified. However, the increased expression of pro-apoptotic miRNAs and the reduced levels of miRNAs in the organ of Corti (oC) linked to aging emphasize their potential relevance in the therapeutic approach to ARHL [19–21]. The regulation of target gene expression by miRNAs occurs through their interaction with the 3’ UTR region of the gene’s mRNA. MiRNAs are crucial for post-transcriptional regulation, interacting with complementary sequences in the 3’ UTR of target mRNAs, which leads to mRNA degradation and inhibition of translation (gene silencing) [22]. In this study, we identified a total of 11 candidate genes through the analysis of RNA sequencing data obtained from individuals with ARHL. Among the identified genes, we focused on the profile of the *FASLG* gene, which is associated with aging. This study aims to explore the role of miRNAs in regulating the mRNA expression of *FASLG*. Specifically, miR-5195 and miR-3941, which modulate *FASLG* expression, could serve as key regulatory factors.

## 2. Materials and methods

### 2.1 Ethical considerations and study participants

All participants included in the study underwent audiometric assessments in a controlled quiet environment using the Interacoustic AC-40 clinical audiometer (Interacoustics, Middelfart, Denmark), following the manufacturer’s instructions. The hearing-healthy group was defined as having an average hearing threshold of ≤ 25dB across the frequencies of 250 Hz, 500 Hz, 1000 Hz, 2000Hz, and 4000 Hz. Conversely, the ARHL group was defined by mean hearing thresholds of ≥ 40 dB across the same frequency range. Participants with a medical history of acute or chronic infections, active infection symptoms, hypertension, diabetes, chronic kidney disease, or other auditory-related disorders were excluded from the analysis. Blood samples were obtained from 12 participants in each cohort, comprising the normal hearing group and the ARHL group, as determined by their audiogram profiles. The gender distribution in the normal hearing group was 5 males to 7 females, whereas the ARHL group consisted of 7 males and 5 females. Additionally, as shown in Table 1, the clinical characteristics of enrolled participants between the ARHL and normal groups are presented.

**Table 1:** Clinical characteristics of enrolled participants between ARHL and normal groups.

	ARHL group (n = 12)	Normal group (n = 12)	P value
Age	68 ± 8.56 years	28 ± 12.89	< 0.01
Sex (M:F)	7:5	5:7	0.543
Average hearing threshold(dB)	67.25 ± 8.23	21.75 ± 4.75	< 0.01
Diabetes	0	0	
Hypertension	0	0	
Chronic renal disease	0	0	

Abbreviations: Age-related hearing loss (ARHL), number (n), Male(M), Female(F), Decibel(dB).

All samples utilized in this study were collected after obtaining written informed consent from all participants. The study was conducted from December 4, 2018, to December 31, 2024, and was approved by the Institutional Review Board of Cheonan Hospital, Soonchunhyang University College of Medicine (IRB No. 2018-10-037-001), in accordance with the Declaration of Helsinki.

### 2.2 Extraction of Peripheral Blood Mononuclear Cells (PBMCs) from human blood samples

Blood samples were collected from 12 individuals in each group, the normal group (28 ± 12.89 years, male = 5:7) and the ARHL group (68 ± 8.56 years, male = 7:5). Total RNA extraction was performed with the QIAamp RNA Blood Mini Kit (Qiagen, Germany) according to the protocols provided by the manufacturer. The RNA concentration was calculated with a Nanodrop spectrophotometer, and the samples were prepared for RT-qPCR analysis. Blood samples were gently mixed in EDTA-coated tubes and layered onto Ficoll-Paque at a 1:1 ratio. The mixture was subjected to centrifugation at 277 rcf for 30 minutes under ambient conditions without the application of a brake, enabling the separation of the peripheral blood mononuclear cell (PBMC) layer. The PBMC fraction was subsequently rinsed with 1 × PBS (pH 7.4) and subjected to centrifugation at 277 rcf for 10 minutes. After discarding the supernatant, the cells were gently resuspended in 1 × PBS to prepare for RNA isolation.

### 2.3 RNA sequencing

RNA samples were processed using the NanoString nCounter Analytic System (NanoString Technologies, Inc., Seattle, WA, USA) [26] in accordance with the manufacturer’s guidelines. For each sample, 5 μL of RNA (100–300 ng) was combined with 8 μL of Master Mix, containing the reporter code set and hybridization buffer, followed by the addition of 2 μL of capture probe set. After centrifugation, the mixture was incubated in a 65°C thermal cycler (Bio-Rad Laboratories, Hercules, California, USA) for 16 hours, ensuring the hybridization time did not exceed 48 hours. The samples were then transferred to the nCounter Master Kit and loaded into a cartridge, which was placed in the preparation station (NanoString Technologies) for processing. The system processed 12 samples per run for approximately 2.5 to 3 hours. After preparation, the cartridge was analyzed using the Digital Analyzer (NanoString Technologies) at 555 fields of view for data collection.

### 2.4 Visualization of RNA seq data

The heatmap was created using the Complex Heatmap package in R [27]. To investigate the functions of DEGs in ARHL, Differentially expressed genes were identified based on a fold-change ≥ 2 and a p-value ≤ 0.05. A total of seven up-regulated and four down-regulated genes were subjected to Gene Ontology (GO) enrichment analysis using the DAVID Bioinformatics Resources 6.8 platform [28]. The GO analysis covered three categories: Biological Processes (BP), Cellular Components (CC), and Molecular Functions (MF) [29].

### 2.5 Quantifying gene expression and differentially expressed gene analysis

Gene expression levels were quantified through an initial normalization step using the geNorm algorithm [30], implemented in nCounter Advanced Analysis version 2.0.115 (NanoString Technologies, Seattle, WA, USA) [31]. Next, genes with differential expressions between the two selected biological conditions were resolved with the default option. The normalized expression values of several hundred selected genes with varying expression patterns were clustered in an unsupervised manner using nCounter Advanced Analysis version 2.0.115, allowing for comparison of expression profiles across samples. nCounter Advanced Analysis version 2.0.115 software allows for the generation of graphs that display changes in gene expression, including fold changes and associated p-values, when comparing two selected samples.

### 2.6 Expression vectors construction

For the transfection experiments, the *FASLG* Human 3’ UTR Clone (#SC210991, OriGene, Rockville, Maryland, USA), miR-5195 (HmiR1468 – MR04, Genecopoeia, Rockville, Maryland, USA), and miR-3941 (HmiR1052 – MR04, Genecopoeia, Rockville, Maryland, USA) were used. The *FASLG* 3′UTR clone was paired with the pMirTarget 3′UTR assay vector, a cloning tool for validating miRNA targets. Luciferase served as the assay reporter, and kanamycin resistance in E. coli DH5α was used for selection. The insert size is 890 base pairs. The construction of miR-5195 and miR-3941 was carried out using the pEZX-MR04 vector, with puromycin employed as the selection marker.

### 2.7 Validation of candidate gene expression

RT-qPCR was performed on whole blood samples from NH and ARHL patients to verify the expression of the genes identified through mRNA sequencing (Table 2). Reverse transcription was performed following the manufacturer’s guidelines with the miScript II RT Kit (Qiagen, Hilden, Germany). For RT-qPCR, the reaction mixture was prepared with 2 µL of 5 × miScript HiFlex Buffer, 1 µL of 10 × miScript Nucleics Mix, 1 µL of miScript Reverse Transcriptase Mix, and 1 µg of RNA template [32]. RNase-free water was added to adjust the total volume to 10 µL. The reactions were carried out using the ABI StepOnePlus™ system (USA). RT-qPCR protocol started with an initial denaturation at 95°C for 10 minutes and proceeded through 40 amplification cycles. Each cycle included 30 seconds of denaturation at 95°C, 30 seconds of annealing at 60°C, and a 1-minute extension phase at 72°C. Primers were designed using Primer 3 software to enable quantitative analysis of target genes [33]. GAPDH was utilized as the internal control, and SYBR Green as-says were employed for the analysis of both target genes and GAPDH primers. Gene expression levels were measured using the Cycle threshold (Ct), and relative expression compared to GAPDH RNA was calculated through the 2 − ΔΔCt method. miRNA target genes were identified by retrieving experimentally validated miRNA-target pairs from miRTarBase [34], while mature miRNA sequences were sourced from the miRBase database [35].

**Table 2:** The primer sequences for RT-qPCR.

	Primer sequences	Product Size (bp)	Annealing Temp (°C)
***FASLG***	5’-CAGCAGCCCTTCAATTACCC-3’ (forward)	231	59
	3’-GCTGTGGTTCCCTCTCTTCT-5’ (reverse)		
***MARCO***	5’-AGACACCCCAAATACTGCGA-3’ (forward)	175	59
	3’-CCCCACCCCTGTATTTCACT-5’ (reverse)		
***NCAM1***	5-’CCGCTGGCAGGAAACAATTC-3’ (forward)	178	59
	3’-TTCACCGTCCTCATCGGTTT-5’ (reverse)		
***NECTIN1***	5’-TTCCAAGTGTCGGGGCTATT-3’ (forward)	299	59
	3’-ACTCCACAGTGCTGCCTTAT-5’ (reverse)		

### 2.8 Cell culture

Cell culture was conducted using two cell lines, HEI-OC-1 and HeLa. HEI-OC-1 was grown in Dulbecco’s Modified Eagle’s Medium (DMEM, WELGENE, Gyeongsan, Republic of Korea) with 10% fetal bovine serum (FBS, Gibco) in culture plates. HeLa was grown in DMEM (WELGENE) with 10% FBS (Gibco) and Penicillin-Streptomycin (P/S, Gibco). The cells were grown at 37°C in a CO_2_ incubator with controlled humidity, maintaining a 5% CO2 atmosphere.

### 2.9 Cell transfection

The House Ear Institute-Organ of Corti 1 (HEI-OC-1) and HeLa cells were plated at a density of 3 × 10⁵ cells per well in 6-well plates and transfected 24 hours post-seeding using Lipofectamine 3000 (Thermo Fisher, USA). Each transfection reaction included 500 ng of miR-5195 and miR-3941 (in pEZX-MR04) targeting the 3’ UTR of *FASLG* in pmiRGLO, along with pmiRGLO for normalization. As controls, off-target control miRNA (Genecopoeia, Rockville, Maryland, USA) was used.

### 2.10 Dual luciferase activity assay

Luciferase activity was assessed in cells co-transfected with the 3′UTR *FASLG* and the pmirGLO control vector using the Dual Luciferase Reporter Assay System (DLR Assay System, Promega, Madison, WI, USA). This method enabled dual luciferase analysis within the pmirGLO-based reporter system. The culture medium was removed 24 hours after transfection, followed by washing the cells with PBS. Next, 200 μL of passive lysis buffer (200 μL per well; Promega, Madison, WI, USA) was introduced into each well, and the plate was gently agitated at room temperature for 15 minutes to facilitate complete cell lysis. The cell lysate was then collected for the dual luciferase assay. A 20 μL portion of the lysate was pipetted into a white opaque 96-well plate (Corning Inc., Corning, NY, USA) for analysis. The luciferase activities of firefly and Renilla were measured in sequence. Relative luciferase activity was determined by normalizing firefly luciferase activity to Renilla luciferase activity, with the pmirGLO vector serving as an internal control to account for differences in cell number and transfection efficiency. In this experiment, we used CmiR-0001-MR04 (Genecopoeia) as an off-target control (Table 3) to verify that the observed effects were specific to the miRNA under investigation, as it shared similar experimental conditions but lacked sequence complementarity to the target gene. Therefore, we used this control to help ascertain whether the observed effects in the experiment were due to the action of the miRNA under investigation.

**Table 3:** The Sequencing Primers for off-target.

miR-control	Sequencing Primers
Off-target	5’-CCGACAACCACTACCTGA-3’(forward)
	3’-CAGAGCGTGTAAGAAGTGC-5’ (reverse)

### 2.11 Network of *FASLG* and miRNAs

To construct the network between the *FASLG* gene and miRNAs, we analyzed context++ score values using the TargetScan website [36]. We identified 35 miRNAs that were common to both the TargetScan and miRTarBase [37] databases. The network was structured using the R (igraph) package. The context++ score is used to evaluate the likelihood that a miRNA will bind to an mRNA and suppress the expression of the target gene. Lower scores indicate a higher probability that the miRNA will effectively target the mRNA [38].

### 2.12 Statistical analysis

Statistical analyses were performed using GraphPad Prism software. Depending on the context, an unpaired two-tailed Student’s t-test or a two-way ANOVA was applied. Results, where appropriate, are presented as the mean ± SEM.

## 3. Results

### 3.1 Different gene expression profiles of the peripheral blood leukocyte of ARHL patients

**Fig 1:**
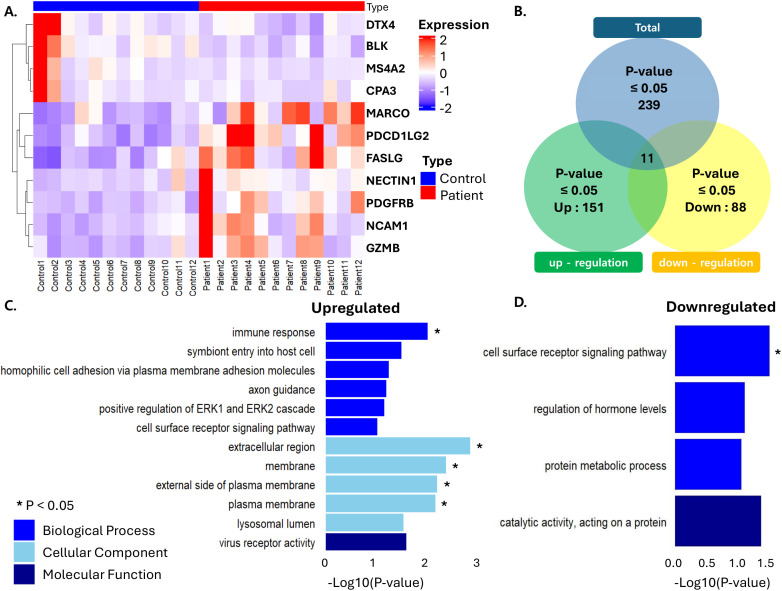
Gene expression profiles & Gene set Enrichment analysis. (A). RNA-sequencing heatmap for the 11 most increased or decreased genes in ARHL. Blue indicates low expression, black represents median expression, and red indicates high expression based on log₁₀(normalized count + 1). Genes were filtered using a threshold of p-value ≤ 0.05 and (|Fold Change|) ≥ 2 Both rows (genes) and columns (samples) were subjected to hierarchical clustering. (B). A Venn diagram depicts the distribution of genes identified as either upregulated or downregulated based on RNA sequencing analysis of serum samples from ARHL patients. A total of 11 genes met the inclusion criteria of a p-value ≤ 0.05 and an absolute fold change (|Fold Change|) ≥ 2. (C). The Functional Gene Ontology analysis of up-regulated genes was conducted using the direct values from DAVID (biological process, cellular component, molecular function). (D). The Functional Gene Ontology analysis of down-regulated genes was conducted using all values from DAVID (biological process, molecular function). Abbreviations used are as follows: Deltex E3 Ubiquitin Ligase 4 (DTX4), BLK Proto-Oncogene (BLK), Membrane Spanning 4-Domains A2 (MS4A2), Carboxypeptidase A3 (CPA3), Macrophage Receptor with Collagenous Structure (*MARCO*), Programmed Cell Death 1 Ligand 2 (PDCD1LG2), Fas Ligand (*FASLG*), Nectin Cell Adhesion Molecule 1 (*NECTIN1*), Platelet-Derived Growth Factor Receptor Beta (PDGFRB), Neural Cell Adhesion Molecule 1 (*NCAM1*), Granzyme B (GZMB), Database for Annotation, Visualization and Integrated Discovery (DAVID).

11 candidate genes exhibiting the most significant differential expression, either upregulated or downregulated with aging, were identified by comparing patients with ARHL to healthy NH controls. This list included seven genes, such as Fas Ligand (*FASLG*), Neural Cell Adhesion Molecule 1 (*NCAM1*), Granzyme B (GZMB), Nectin Cell Adhesion Molecule 1 (*NECTIN1*), Platelet-Derived Growth Factor Receptor Beta (PDGFRB), Macrophage Receptor with Collagenous Structure (*MARCO*) and Programmed Cell Death 1 Ligand 2 (PDCD1LG2), which demonstrated a minimum of 2-fold up-regulation in the ARHL group when contrasted with the NH group, with adjusted p-values below 0.05 in both populations. Four genes, such as BLK Proto-Oncogene (BLK), Membrane Spanning 4-Domains A2 (MS4A2), Carboxypeptidase A3 (CPA3) and Deltex E3 Ubiquitin Ligase 4 (DTX4), exhibited a minimum of two-fold down-regulation in the ARHL group when compared with the NH group, with adjusted p-values less than 0.05 consistently across both populations (Fig 1A). The Venn diagram illustrates the number of genes that were differentially expressed, either upregulated or downregulated, as determined from RNA sequencing data obtained from the serum of ARHL patients (Fig 1B). The GO analysis in Fig 1C was conducted using the seven up-regulated genes. The results for the Biological Processes (BPs) category indicate involvement in immune response, symbiont entry into host cells, homophilic cell adhesion via plasma membrane adhesion molecules, axon guidance, positive regulation of the ERK1 and ERK2 cascade, and cell surface receptor signaling pathways. For Cellular Components (CCs), the analysis shows participation in membrane structures, including the external side of the plasma membrane, the plasma membrane itself, and the lysosomal lumen. The Molecular Function (MF) analysis points to involvement in virus receptor activity. The pathways showing a statistically significant pathway were immune response, extracellular region, membrane, external side of the plasma membrane, and plasma membrane (*p < 0.05). In contrast, the GO analysis in Fig 1D was based on four downregulated genes. The BP results suggest participation in the cell surface receptor signaling pathway, regulation of hormone levels, and protein metabolic processes. The CC results point to catalytic activity, specifically acting on proteins. The pathway showing a statistically significant difference was the cell surface receptor signaling pathway (*p < 0.05).

### 3.2 Validation of target genes by RT-qPCR

Using RT-qPCR, gene sequencing results for selected Differentially Expressed Genes (DEGs) were confirmed with whole blood (WB) samples of ARHL. Among the Whole blood differentially expressed genes (WB-DEGs), four genes showing correlation with RNA sequencing data were found: Fas Ligand (*FASLG*), Neural Cell Adhesion Molecule 1 (*NCAM1*), Nectin Cell Adhesion Molecule 1 (*NECTIN1*), Macrophage Receptor with Collagenous Structure (*MARCO*). The four genes were found to be significantly upregulated in whole blood from ARHL patients compared to the NH group. Although all four genes showed statistically significant differences, we selected the *FASLG* gene because it exhibited the highest statistical significance (**P < 0.01).

**Fig 2:**
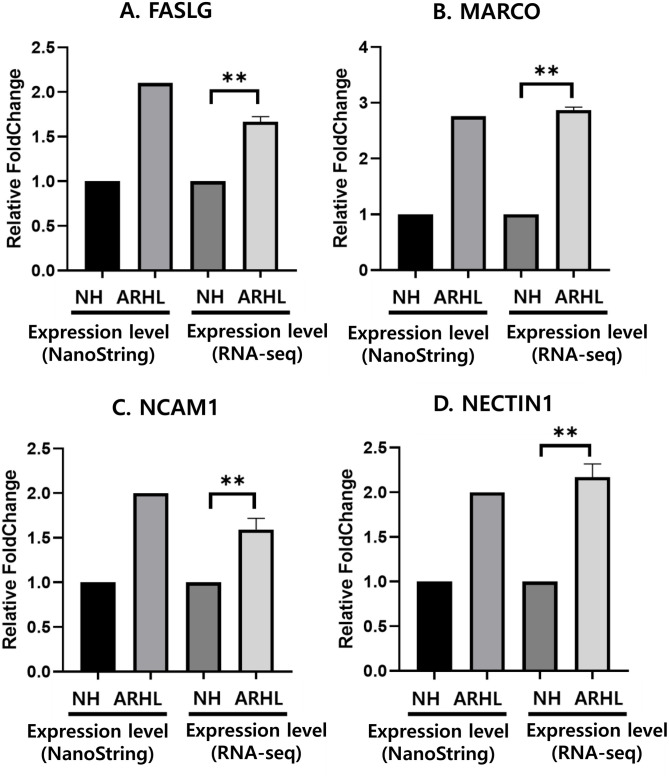
Validation of target genes by RT-qPCR with whole blood samples. NH and ARHL represent whole blood of healthy NH and patients with ARHL, respectively. GAPDH served as the internal control for normalization. The expression levels of *FASLG*, *MARCO*, *NCAM1*, and *NECTIN1* were normalized relative to GAPDH (** p < 0.01). The expression of all genes (Fig 2A-2D) was confirmed using RT-qPCR comparing the NH group (n = 12) and the ARHL group (n = 12). Abbreviations used are as follows: Normal hearing group (NH), Age-related hearing loss (ARHL), Fas Ligand (*FASLG*), Macrophage Receptor with Collagenous Structure (*MARCO*), Neural Cell Adhesion Molecule 1 (*NCAM1*), Nectin Cell Adhesion Molecule 1 (*NECTIN1*).

Among the four genes that were consistently upregulated in ARHL patients (*FASLG*, *NCAM1*, *MARCO*, and *NECTIN1*), *FASLG* was prioritized for further validation based on its strong association with immune-mediated apoptosis and its previously reported role in age-related immune dysfunction. In addition, *FASLG* was predicted to be a potential target of multiple miRNAs, including miR-5195 and miR-3941, making it a suitable candidate for exploring post-transcriptional regulation mechanisms in ARHL (Fig 2).

### 3.3 Inverse correlation of *FASLG* and target miRNAs

**Fig 3:**
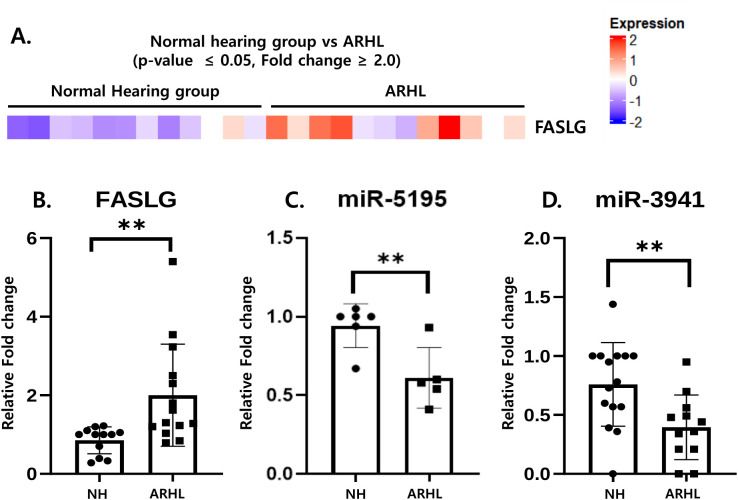
Inverse correlation of *FASLG* and target miRNAs in the whole Blood of NH and ARHL. (A). Heat map illustrating differential expression data from RNA-Seq analysis. Heatmap of *FASLG* gene expression, represented as fold changes in mRNA levels, was generated for the ARHL (n = 12) and NH (n = 12); (B). Expression of *FASLG* was analyzed using RT-qPCR and confirmed through WB (NH: n = 12, ARHL: n = 14). (C). Expression of miR-5195 was detected using RT-qPCR in WB (NH: n = 6, ARHL: n = 5). (D). Expression of miR-3941 was detected using RT-qPCR in WB (NH: n = 14, ARHL: n = 12). U6 snRNA was used as an internal control (** p < 0.01). Abbreviations used are as follows: Normal hearing group (NH), Age-related hearing loss (ARHL), Whole blood (WB), Fas ligand (*FASLG*).

Candidate gene expression profiles were analyzed using RNA sequencing data from the serum of ARHL patients. A heatmap depicting the expression of the *FASLG* gene based on mRNA level changes in the ARHL and NH groups is shown. RNA-Seq differential expression analysis revealed that *FASLG* was up-regulated in ARHL patients (n = 12) (Fig 3A). Although its structure is not fully characterized, the interaction between Fas and FasL involves the binding of trimeric *FASLG* to Fas, resulting in the formation of a signaling-active trimer that triggers the assembly of a death-inducing signaling complex (DISC) with intracellular proteins. Our findings indicated that immune responses were amplified throughout the organism, resulting in the depletion of specific blood cell populations (Fig 3B). The RT-qPCR analysis presented in Fig 3B shows an increased fold change in *FASLG* expression in the ARHL patient group compared to the NH group. As shown in Fig 3C and 3D, the expression levels of miR-5195 and miR-3941 were significantly reduced in the ARHL patient group compared to the NH group. We selected miR-5195 and miR-3941 for further investigation based on their predicted binding to the 3′UTR of FASLG and their differential expression patterns in the RNA-seq data. Unlike previously reported miRNAs such as miR-21, these candidates had not been well-characterized in the context of FASLG regulation or age-related hearing loss. Thus, we prioritized them to explore novel regulatory pathways that may underlie the observed upregulation of FASLG in ARHL.

### 3.4 Down-expression of *FASLG* by a target of miR-3941 and 5195-5p *in vitro*

**Fig 4:**
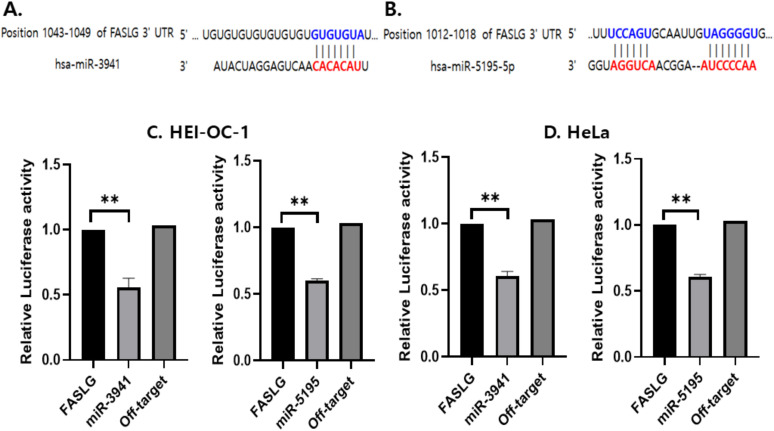
Findings from the dual luciferase reporter assay assessing miRNAs and their target genes. (A). Identification of 3′-UTR sequences in the *FASLG* gene predicted to include binding sites for miR-3941; (B). Identification of 3′-UTR sequences in the *FASLG* gene predicted to harbor binding sites for miR-5195-5p; (C). Dual luciferase reporter assay results for miR-3941, miR-5195, and their corresponding target 3’-UTR of *FASLG* in HEI-OC-1 cells; (D). Dual luciferase assay results of miR-3941, miR-5195 and their corresponding target 3’-UTR of *FASLG* in HeLa cell; The significant differences between *FASLG* and the miRNA target group are represented as ** p < 0.01. Abbreviations used are as follows: Fas ligand (*FASLG*), House Ear Institute-Organ of Corti 1 (HEI-OC-1).

To identify potential regulators of *FASLG* expression in ARHL, miRNAs targeting *FASLG* were discovered through bioinformatics tools such as miRTarBase, miRBase, and TargetScan. The validation of target miRNAs was performed using luciferase assays. A human *FASLG* vector (pmirGLO) containing a miRNA binding site was utilized, with the firefly luciferase gene fused downstream (Fig 4). Fig 4A shows that the *FASLG* 3′UTR contains a binding site for miR-3941. Fig 4B illustrates that the *FASLG* 3′UTR has two binding sites for miR-5195-5p. In HEI-OC-1 cells, luciferase activity was reduced following co-transfection with miR-3941, miR-5195, and *FASLG* (Fig 4C). Similarly, in HeLa cells, luciferase activity decreased after co-transfection with miR-3941, miR-5195, and *FASLG* (Fi 4D). The reduction in luciferase activity suggests that miR-3941 and miR-5195 bind to the target site and downregulate *FASLG* expression. Notably, off-target miRNAs did not negatively impact the predicted binding sites of miR-3941 and miR-5195.

### 3.5 Network connection between *FASLG* and miRNA & pathway

**Fig 5:**
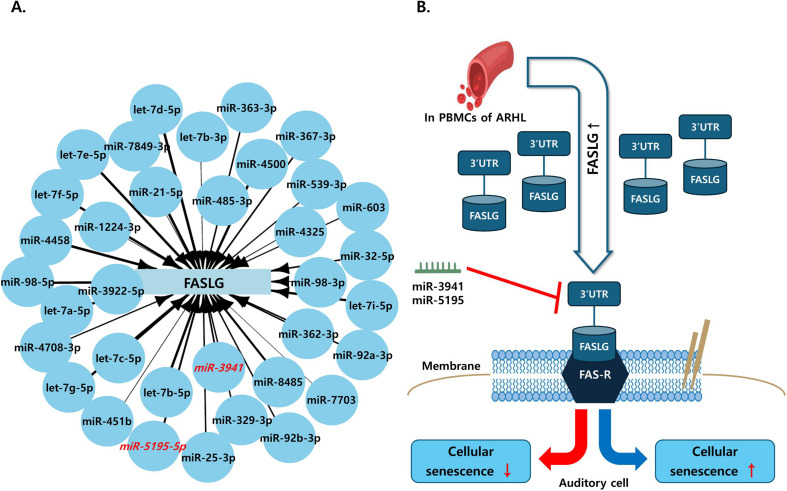
The network and pathway interactions of *FASLG* and miRNAs. (A). The light blue rectangular node represents the *FASLG* gene, while the light blue circular nodes represent the 35 selected miRNAs. The nodes with red text indicate the two miRNAs of interest, miR-3941 and miR-5195-5p. The arrows connecting *FASLG* to the miRNAs indicate that the thickness of the arrow corresponds to the context++ score of the miRNA, with thicker arrows representing lower scores. (B). We utilized blood samples from individuals with ARHL and found that A notable rise in *FASLG* expression was observed. Additionally, we identified miR-3941 and miR-5195 as potential regulators of *FASLG* expression. These miRNAs may bind to the 3’ UTR of the elevated *FASLG*, leading to down-regulation of gene expression. Abbreviations used are as follows: Fas ligand (*FASLG*), Age-related hearing loss (ARHL), Fas receptor (FAS-R), 3’untranslated region (3’UTR).

To demonstrate which miRNAs are involved in *FASLG* regulation, we selected 35 miRNAs identified from TargetScan and miRtarBase (Fig 5A). Among the 35 candidate miRNAs, miR-5195 and miR-3941 were prioritized for experimental validation due to their strong predicted binding to the 3′UTR of FASLG and consistent downregulation in RNA-seq analysis. Previously known regulators such as miR-21 were not selected, as they did not show meaningful expression changes or high predictive scores in our data. Although limited literature exists for miR-5195 and miR-3941 in the context of ARHL, this gap underscores the novelty of our findings.

We utilized the context++ score obtained from TargetScan, where lower values indicate a greater inhibitory effect on mRNA, thereby reducing the expression of the target gene. Based on this principle, we constructed a network, where thicker connection lines indicate lower context++ scores. miR-3941 and miR-5195-5p, which were validated in the previous method, are highlighted in red, allowing us to predict the expression levels of the miRNAs of interest, miR-3941 and miR-5195-5p. The cellular senescence pathway summarizes the action of miRNAs in response to the upregulation of *FASLG*, one of the genes identified as being associated with ARHL (Fig 5B). Our study suggests that miR-3941 and miR-5195 are key regulators of *FASLG* expression, potentially in-fluencing cellular senescence.

## 4. Discussion

Hearing loss can be caused by various agents, such as aging, ototoxic drugs, noise, virus infection, and genetic mutations. However, there is currently no definitive treatment for hearing disorder, especially ARHL, and various clinical trials or re-searches are ongoing. Through RNA sequencing, the present study showed that the overexpression of the *FASLG* gene may influence the development of ARHL, and miR-5195 and miR-3941 could reduced the expression of *FASLG* gene. These microRNAs represent the first report in the field of ARHL research and could be one of the potential approaches in ARHL treatment.

While this study compared ARHL patients with younger, normal-hearing individuals, we acknowledge that the observed differences may be influenced by general aging rather than hearing loss alone. Previous studies have shown that aging is associated with changes in immune-related gene expression, including *FASLG*, *NCAM1*, and *MARCO*, which may also contribute to normal auditory aging. Therefore, the distinction between age-related changes and ARHL-specific alterations requires further investigation, ideally using age-matched cohorts with different hearing statuses.

The inner ear elicits an immune response that interacts with the systemic immune system, potentially leading to cochlear degeneration and ARHL [39]. These inflammatory process in cochlea could be caused by inner ear macrophages recruited form blood-borne monocytes from bone marrow myeloid precursors, but may be resident [40,41]. Macrophages are a key part of immune response and inflammatory process in cochlea [5,6]. Several papers described that macrophages were present in adult cochlea, including the organ of Corti, spiral ligaments of lateral wall, and have a close interaction with the stria microvasculature, a critical component of the blood-labyrinthine barrier that regulates cochlear homeostasis [42–44]. Additionally, macrophage activation and changes in the cochlear immune system can lead to chronic inflammation that contributes to ageing in the cochlear microenvironment. This process plays a central role in degeneration and dysfunction of cochlear cells in presbycusis [45]. Therefore, the present study focused on changes in gene expression in peripheral blood leukocytes of ARHL patients and showed that these changes in the systemic immune system may be associated with ARHL.

Although cochlear tissues would provide more direct insights into ARHL-related molecular changes, they are not easily accessible in living human subjects. Therefore, this study utilized PBMCs as a practical and biologically relevant surrogate, based on growing evidence that systemic immune dysregulation and chronic low-grade inflammation (“inflammaging”) are closely linked to age-related sensory decline [46]. PBMCs have been previously used as an accessible source for studying transcriptomic changes related to neurodegeneration, immune aging, and sensory disorders, and thus serve as a meaningful model for evaluating immune-related gene expression in ARHL [47,48].

*FASLG*, *MARCO*, *NCAM1*, and *NECTIN1* genes are closely related to the immune system, each playing a pivotal role in various immune processes. *FASLG* promotes T cell apoptosis, which contributes to regulating autoimmune responses [49]. *MARCO* is crucial in macrophage function, facilitating the removal of pathogens and particles, thus supporting the innate immune response [50]. Additionally, *MARCO* expression has been linked to reduced survival rates in pancreatic cancer, where it plays a significant role in tumor progression [51]. *NCAM1* is involved in regulating interactions between T cells and B cells, influencing immune surveillance [52]. Furthermore, *NCAM1* is associated with immune evasion in acute myeloid leukemia (AML), where it promotes leukemogenesis and drug [53,54]. Lastly, *NECTIN1* plays a crucial role in modulating immune responses by interacting with various immune cells, and its ex-pression is linked to disease progression in conditions such as hepatocellular carcinoma [55]. Especially, *FASLG*, a member of the tumor necrosis factor superfamily, is primarily responsible for inducing apoptosis through its interaction with the Fas receptor [56]. This transmembrane protein exhibits a dual role, functioning in both apoptosis and immune regulation. In immune regulation, *FASLG* is linked to T cell activation, where it binds to the Fas receptor on other T cells, triggering apoptosis and maintaining immune homeostasis [57]. Apoptosis is facilitated by the Fas/FasL system, primarily regulating the extrinsic pathway of apoptosis [58]. During this process, *FASLG* promotes the formation of the DISC, which recruits adapter proteins such as FADD and procaspase 8 or 10, ultimately leading to apoptosis [59]. Therefore, the present study demonstrated that *FASLG* expression was significantly up-regulated in the ARHL group, and these changes might be associated with alterations of systemic immune response related to ageing. Although this study identified the upregulation of *FASLG* and its regulation by miR-5195 and miR-3941, we did not directly assess the functional consequences of *FASLG* modulation on cochlear cell viability or auditory function. While HEI-OC1 cell-based assays provide preliminary insights, further validation through animal models of ARHL, including *FASLG* overexpression or knockdown, auditory brainstem response (ABR) testing, and TUNEL staining, will be essential to clarify the causal relationship between *FASLG* activity and auditory decline. In addition, we recognize that the use of only HEI-OC1 cells limits the physiological relevance of our findings. Although this model is established and widely used in ARHL research, it cannot fully replicate the complex cochlear environment. Thus, our results should be considered preliminary molecular evidence. To strengthen these findings, future studies incorporating cochlear explants or in vivo models will be essential to validate the mechanistic role of FASLG and its regulation by miRNAs in the auditory system. These studies will also address functional outcomes—such as changes in auditory thresholds—using auditory brainstem response (ABR) testing, TUNEL assays, and targeted gene manipulation. Finally, given the inherent difficulty of fully separating aging-related effects from hearing loss, follow-up work using ex vivo and animal models will be important to better control for these confounding factors.

Ultimately, demonstrating hearing recovery through in vivo modulation of FASLG expression—such as knockdown or overexpression in age-related hearing loss (ARHL) animal models like C57BL/6 mice, followed by auditory brainstem response (ABR) testing—will be essential to provide definitive evidence for its functional role and therapeutic potential in ARHL. While most prior studies have primarily relied on animal models, our approach, which integrates molecular profiling of human-derived biospecimens from ARHL patients with in vitro validation in HEI-OC1 cells, offers a complementary perspective that may help accelerate the identification of therapeutic targets for hearing loss. Notably, this study is the first, to our knowledge, to compare *FASLG*, its associated genes, and regulatory miRNAs in human-derived samples related to ARHL. Our findings lay the groundwork for future functional studies aimed at evaluating *FASLG* as a potential therapeutic target for ARHL.

Furthermore, although we proposed roles for miR-5195 and miR-3941 in immune modulation and aging-related processes, our study did not directly examine downstream pathways such as cytokine secretion or macrophage activation. Future studies using cytokine assays and flow cytometry will be conducted to elucidate the functional roles of these miRNAs in ARHL pathology.

Additionally, the present study explored post-transcriptional regulation mechanisms, specifically focusing on how miRNAs bind to the 3’ UTR of *FASLG*’s mRNA, which plays a crucial role in fine-tuning gene expression at the post-transcriptional level.

MiRNA expression regulation is crucial for controlling target gene expression, as well as for processes such as proliferation, differentiation, and apoptosis [60]. MiRNAs are small noncoding RNAs that are crucial for controlling gene expression [61]. MiRNA primarily regulates gene expression at the post-transcriptional stage by interacting with mRNA, either suppressing gene expression or promoting mRNA degradation [62]. There are several studies linking the regulation of *FASLG* expression to the upregulation or downregulation of miRNAs. For instance, miR-21 is one of the key miRNAs that regulate *FASLG* expression and plays a role in promoting tumor cell invasiveness and proliferation, particularly in cancer. Overexpression of miR-21 suppresses *FASLG* expression, reducing apoptosis and contributing to cancer progression [63,64]. Zhang et al. reported that miR-149-5p overexpression inhibits *FASLG*, leading to the suppression of apoptosis. Conversely, blocking miR-149-5p results in increased *FASLG* expression, which in turn promotes apoptosis in leukemia cells [65]. Regis et al. suggest that miR-24-3p reduces the expression of *FASLG* in their study on the relationship between miR-24-3p and *FASLG* [66]. As of current research, no specific studies have been identified that directly show miR-5195 and miR-3941 regulating the expression of *FASLG*. However, both miR-5195 and miR-3941 have been reported to play significant roles in regulating apoptosis in cancer cells by targeting the IGBP1 gene [67]. Additionally, miR-5195 is known for its tumor-suppressive function and its ability to inhibit cancer cell proliferation [68,69]. This study is the first to demonstrate that miR-5195 and miR-3941 regulate the target gene *FASLG* in the context of hearing research. We found that the expression of *FASLG* could be controlled by miR-3941 and miR-5195 in HEI-OC-1 cells. However, the specific roles of miR-5195 and miR-3941 on the protective mechanisms of ARHL need further investigation in an animal model.

While HEI-OC1 cells are widely used as an *in vitro* model for auditory research, they do not fully recapitulate the structural and cellular complexity of the cochlear environment. Therefore, the functional role of FASLG and its regulation by miR-5195 and miR-3941, as demonstrated in this study, should be further validated in physiologically relevant models. Future studies using cochlear explants or in vivo ARHL animal models will be necessary to confirm the translational applicability of these findings.

A major limitation of this study is the absence of direct analysis using cochlear tissue, which limits the ability to confirm the physiological relevance of the observed FASLG and miRNA interactions in the native auditory environment. Although HEI-OC1 cells are widely used as an in vitro model for auditory research, they cannot fully replicate the complex cellular architecture and functionality of the cochlea. To reduce the influence of systemic factors on hearing, individuals with comorbidities known to affect auditory function, such as diabetes and hypertension, were excluded from the study. However, to validate the biological significance of the findings, future research using cochlear explants or in vivo models of age-related hearing loss, such as C57BL/6 mice, will be necessary.

Research on drug development for the prevention and treatment of ARHL is progressing in various directions. Smith et al. examine the effects of molecular and cellular aging pathways on the progression of ARHL. It highlights the significant roles of pathways such as AMPK, mTOR, and insulin-like growth factor 1 (IGF-1) in relation to the progression of ARHL and discusses the potential of targeting these pathways for therapeutic intervention. Additionally, drugs that modulate anti-aging pathways like sirtuins are proposed as major targets for ARHL treatment [70]. Jones et al. explore recent developments in nanotechnology and biotechnology for treating ARHL. It highlights the potential of bile acid-based nanotechnology to enhance drug delivery, offering a promising approach for preventing and treating ARHL. Additionally, it notes that antioxidants and anti-inflammatory agents can play a crucial role in preventing auditory damage, while growth factors like IGF-1 may contribute to auditory nerve protection [71]. Audion Therapeutics, a Netherlands-based clinical-stage biopharmaceutical company, has reported results from a clinical trial of the gamma-secretase inhibitor LY3056480 aimed at treating sensorineural hearing loss (SNHL) [72]. This drug was developed to promote the regeneration of hair cells in the inner ear with the goal of restoring hearing. Initial clinical trials showed positive outcomes, including improved speech recognition in noisy environments for some patients, and confirmed the drug’s safety. However, the expected hearing recovery effects were not observed in all patients, indicating the need for further research [73]. The present study suggested that miRNAs could serve as potential treatment methods or preventive tools for mitigating ARHL.

Further research is needed on the four genes identified, including *FASLG*, as well as on the miRNAs that interact with these genes to regulate their expression. This additional research will help to elucidate the mechanisms of gene expression regulation and the role of miRNAs in these processes. This study faced limitations due to the small sample size and reliance on *in vitro* data. Future studies involving animal models are necessary to confirm and build upon these results.

Due to limitations in clinical recruitment and sample availability, we were unable to increase the cohort size in this study. While this may reduce statistical power, our *in vitro* experiments were performed in triplicate and yielded consistent results. In future studies, we plan to include a larger and independently recruited cohort to enhance statistical reliability and generalizability.

Additionally, age-related variables were not fully controlled, which should be considered when interpreting the findings. Despite these limitations, the use of human whole blood samples and other human-derived materials lends significant academic value to this research. This study compared peripheral blood mononuclear cell (PBMC) transcriptomes between younger individuals with normal hearing and older individuals with age-related hearing loss. While sex was controlled and participants with a history of smoking, hypertension, or diabetes were excluded, age-matching was not performed, which may limit the ability to isolate molecular signatures specific to ARHL. Therefore, the observed gene expression changes may reflect broader aging-related immune alterations rather than changes uniquely linked to hearing impairment.

Notably, some of the differentially expressed genes identified in this study, such as *FASLG*, *NCAM1*, and *MARCO*, have been previously implicated in age-related expression shifts in immune cells and are associated with immune senescence. These genes may play a role in both pathological and physiological auditory aging. To distinguish age-related changes from those specifically associated with ARHL, future studies incorporating age-matched groups with varying hearing status will be essential. Such designs will provide deeper insights into the molecular underpinnings of ARHL and contribute to the identification of potential biomarkers or therapeutic targets.

In addition, the relatively small sample size in our study limits the statistical power and generalizability of the findings. As noted, comparing younger individuals with normal hearing to older individuals with ARHL may introduce confounding effects due to the combined influence of age and hearing status. In ARHL research, aging and hearing decline are inherently interconnected, making it challenging to completely disentangle their effects.

To address this in future investigations, we plan to employ more rigorous study designs, including the use of age-matched control groups with varying hearing thresholds and the application of multivariate analytical models, to better isolate ARHL-specific molecular changes. These limitations may reduce the strength of the evidence and highlight the need for larger, more stratified cohorts in future investigations.

The study highlights a significant inverse correlation between the expression levels of *FASLG* and miR-5195 and 3941, suggesting their potential involvement in the development of hearing loss. Additionally, it offers valuable insights into how miR-5195 and 3941 may contribute to auditory dysfunction.

## 5. Conclusions

The overexpression of the *FASLG* gene may be associated with the development of ARHL, and the inhibitory role of miR-5195 and miR-3941 could be a key factor in the prevention or protection against ARHL. The analysis of single nucleotide polymorphisms (SNPs) associated with ARHL provides valuable insights and potential directions for future research in this field.
